# PROX1, a Key Mediator of the Anti-Proliferative Effect of Rapamycin on Hepatocellular Carcinoma Cells

**DOI:** 10.3390/cells11030446

**Published:** 2022-01-27

**Authors:** Sora Kwon, Kiwon Ban, Young-Kwon Hong, Jung-Suk Sung, Inho Choi

**Affiliations:** 1 Department of Pharmaceutical Engineering, Hoseo University, Asan 31499, Korea; kwon_sora_@naver.com; 2 Department of Biomedical Sciences, City University of Hong Kong, Hong Kong 999077, China; Ban.KW@cityu.edu.hk; 3 Department of Surgery, Norris Comprehensive Cancer Center, Keck School of Medicine, University of Soutern California, Los Angeles, CA 90033, USA; young.hong@usc.edu; 4 Department of Life Science, Dongguk University, Goyang 10326, Korea; sungjs@dongguk.edu

**Keywords:** hepatocellular carcinoma, MTOR, rapamycin, PROX1, antiproliferation

## Abstract

The MTOR signal is known to be activated in various cancer cells including hepatocellular carcinoma (HCC) cells. Rapamycin, a specific inhibitor of MTOR, has been widely used as an immunosuppressant in organ transplant patients, and its clinical application has been recently expanded to cancer therapy. In this study, the anti-proliferative effect of rapamycin was investigated in four different HCC cell lines. Rapamycin effectively inhibited the proliferation of Huh7 or Hep3B, but not that of HepG2 or SNU3160 cells. Interestingly, rapamycin increased Prospero-related homeobox 1 (PROX1) expression at the protein level, but did not affect its transcript in Huh7 as well as Hep3B cells. Moreover, immunoprecipitation assays showed that PROX1 ubiquitination was downregulated by rapamycin. Furthermore, PROX1 over-expression or siRNA knock-down in Huh7 and Hep3B cells reduced or increased proliferation, respectively. The effect of PROX1 over-expression on the sensitivity to rapamycin was not synergistic, but the effect of MTOR inhibition on cell proliferation was diminished by PROX1 siRNA. Finally, Huh7 cells were inoculated into the flanks of nude mice and rapamycin was injected daily for 14 days. The xenograft volume was decreased and PROX1 expression was increased by rapamycin. These results indicate that PROX1 plays a key role in the anti-proliferative effect of rapamycin and suggest that the increased PROX1 by MTOR inhibition can be used as a useful marker for predicting whether HCC cells can be affected by rapamycin.

## 1. Introduction

Hepatocellular carcinoma (HCC) is one of the most common malignant cancers [1] and the third leading cause of cancer-related deaths worldwide [2]. However, only 30% of HCC patients can be treated by liver resection, transplantation, or local ablation, mostly owing to its heterogeneous etiology. Regarding chemotherapy, a small number of drugs are available to treat HCC patients [3]. Unfortunately, recent clinical trials have shown that sorafenib is not very effective for all HCC patients. The results from the SHARP (Sorafenib HCC Assessment Randomized Protocol) trial demonstrated that the overall survival (OS) prolongation of HCC patients receiving sorafenib was only 2.8 months, and that objective tumor response occurred in only a small proportion of patients (0.6% to 2%) [4]. Recently, lenvatinib, a multikinase inhibitor, was approved for the treatment of advanced or unresectable HCC patients who had not received prior systemic therapy. However, OS proportions of patients treated with Lenvatinib were only 18%. Thus, it is highly required to identify molecular markers that predict the patients likely to benefit from sorafenib or lenvatinib chemotherapy [5,6].

Recently, it was reported that the mammalian target of rapamycin (MTOR, a serine- threonine kinase of the phosphatidylinositol (PI) kinase-related protein kinase family [7]) is activated in various carcinoma cells, including breast cancer [8], ovarian cancer [9], and HCC [10]. MTOR regulates cell growth and proliferation by controlling translation of several proteins, such as cyclin dependent kinase inhibitor p27kip1, retinoblastoma protein, cyclin D1, c-myc, or STAT3 [11]. The MTOR signal can be activated by various stimuli such as growth factors, nutrients through receptor tyrosine kinase (RTK), phosphatidyl inositol 3 kinase (PI3K), and the Akt/PKB signaling cascade [12]. The MTOR signal elicits its effect by binding to the cytosolic immunophilin FKBP12 (FK506 binding protein, 12 kDa) [13]. Owing to its activation in cancer cells, specific inhibitors of MTOR including rapamycin have been considered as attractive reagents for cancer therapy [11]. Rapamycin, also known as sirolimus, shows its unique inhibitory effects by binding to MTOR and has been used extensively in transplantation to prevent organ rejection [14,15], and recently, its application has expanded to cancer therapy [16].

It was recently reported that Prospero-related homeobox 1 (PROX1), an important transcription factor responsible for the development of several tissues such as lymphatics or liver [17,18,19], contributes to cancer development and progression. PROX1 has been ascribed tumor suppressive and oncogenic properties in different cancer types [20]. Liu et al. [21] reported that PROX1 promotes HCC cell proliferation, but it was also demonstrated that PROX1 can inhibit the proliferation of hepatocellular or oral carcinoma cells [22,23].

Despite the significant roles of MTOR and PROX1 in the proliferation of carcinoma cells, few studies have investigated the relationship between these two factors. Thus, we examined the anti-proliferative effect of rapamycin on HCC and its relation to PROX1 expression in four different HCC cell lines: Huh7, Hep3B, HepG2, and SNU3160. Interestingly, we found that rapamycin reduced the proliferation of Huh7 or Hep3B cells, but not that of HepG2 or SNU3160 cells. Moreover, rapamycin significantly increased PROX1 expression in Huh7 and Hep3B cells by increasing the half-life of PROX1 protein by reducing its ubiquitination without affecting PROX1 transcript.

Subsequent functional studies using PROX1 knock-down or over-expression showed that PROX1 is a critical mediator of the anti-cancer effect of rapamycin. Furthermore, in an in vivo study using Huh7 xenograft, rapamycin reduced xenograft volume and increased PROX1 expression in tumors. Together, these results indicate that PROX1 plays an important role in the anti-proliferative effect of rapamycin and suggest that PROX1 protein level might be a useful marker for anti-cancer therapy of rapamycin in HCC.

## 2. Materials and Methods

### 2.1. HCC Cell Lines and Reagents

Human HCC cell lines, Huh7, Hep3B, HepG2, and SNU3160 (purchased from the Korean Cell Line Bank, Seoul National University, Seoul, Korea), were cultured in Dulbecco’s modified Eagle medium (DMEM) with 10% fetal bovine serum (FBS), 5% penicillin-streptomycin, and 5% sodium pyruvate, in an atmosphere containing 5% CO_2_ at 37 °C. Cells were treated with or without rapamycin (10 nM, LC Laboratories, Woburn, MA, USA) in low serum media (1% FBS) for 48 h, unless otherwise indicated. Cells were transfected with the small interfering RNA (siRNA) against PROX1 (forward: 5′-GCAAAGAUGUUGAUCCUUCTT-3′ and reverse: 5′-GAAGGAUCAACAU-CUUUGCTT-3′) for knock-down experiment or the over-expression system of PROX1 (human PROX1 coding sequence in pcDNA3, kindly provided by Prof. Y-K Hong, University of Southern California) using Lipofactor-2000 (Aptabio, Korea). To measure PROX1 protein stability, cells were treated with cycloheximide (CHX, Sigma-Aldrich, San Diego, CA, USA) for up to 6 h after incubation for 24 h with or without rapamycin.

### 2.2. Cell Proliferation Assay

Cell proliferation was analyzed using the Premixed WST-1 Cell Proliferation Assay kit (TaKaRa, Tokyo, Japan), as described previously [24]. In brief, cells (Huh7, 2 × 10^4^ cells/well; HepG2, Hep3B, and SNU3160 cells, 3 × 10^4^ cells/well) were seeded in 24-well plates (SPL Inc., Pyeongtaek, Korea) and cultured overnight. Media were then replaced with fresh conditional media (1% FBS) containing rapamycin, and cells were allowed to grow for 48 h. WST-1 reagent was then added to each well and cells were incubated for an additional 4 h. Optical absorbance was measured at 450 nm using a microplate reader (Molecular Devices, San Jose, CA, USA), and cell counts were estimated using a standard curve.

### 2.3. Immunofluorescence Microscopic Analysis

Cells (1 × 10^5^ cells per well) were plated in six-well culture plates (SPL Inc.,Pyeongtaek, Korea). The next day, cells were rinsed with ice-cold PBS and fixed with 4% paraformaldehyde for 20 min at room temperature followed by permeabilization with 0.2% Triton X-100 in PBS. The cells were subjected to immunofluorescence staining with anti-PROX1 antibody (1:300, kindly provided by Prof. Y-K Hong, University of Southern California) [25] for 1 h at room temperature, washed three times with cold PBS for 10 min, and then incubated with Alexa Fluor 555-labeled anti-rabbit secondary antibody (1:800) (Santa Cruz Biotechnology, San Diego, CA, USA) at room temperature for 1 h. Cell images were observed and captured using fluorescence microscopy (Leica, Wetzlar, Germany).

### 2.4. Reverse Transcription-Polymerase Chain Reaction

PROX1 transcript expressions were analyzed by semi-quantitative RT-PCR using human PROX1-specific primers (forward: 5′-GCAAGTTGTGGACACTGTGG-3′, reverse: 5′-GGCAGACTGGTCAGAGGAGTT-3′) and *β-actin*-specific-primers (forward: 5′-CTGGGACGACATGGAGAAAA-3′, reverse: 5′-AAGGAAGGCTGGAAGAGTGC-3′) for quantitative comparison. Briefly, total RNA was isolated from cells using TRIzol reagent (Life Technology, Carlsbad, CA, USA) and reverse-transcribed using MMLV reverse transcriptase (Enzynomics, Daejeon, Korea) and 15 mer oligo-dT (Enzynomics, Daejeon, Korea). Prepared cDNAs were used as templates and PCR was performed according to the following protocols: (1) 94 °C for 5 min; (2) 26 cycles at 94 °C for 30 s, 58 °C for 30 s, and 72 °C for 1 min; and then (3) 72 °C for 10 min before cooling to 4 °C. PCR products (10 µL) were loaded on a 1.5% agarose gel containing ethidium bromide and visualized under UV light. Quantitative real-time PCR analysis was performed using a Step OnePlus Real-Time PCR System instrument (Applied Biosystems, Warrington, UK) using Power SYBR^®^ Green PCR Master Mix (Applied Biosystems, Warrington, UK) using the following protocol: 50 °C for 10 min, 95 °C for 2 min, 40 cycles of 95 °C for 10 s, and final extension for 30 s at 55 °C.

### 2.5. Protein Extraction and Western Blot Analysis

Cells (2 × 10^5^) were plated in 60 mm dishes, treated with the indicated concentrations of rapamycin for 0~48 h, and then lysed using RIPA buffer (50 mM Tris-HCl [pH 7.4], 150 mM NaCl, 1% Nonidet P-40, 0.1% sodium dodecyl sulfate [SDS], and 0.5% sodium deoxycholate) containing protease and phosphatase inhibitors (Sigma-Aldrich, San Diego, CA, USA). Proteins were quantified using a Bradford Protein Assay kit (Pierce Biotechnology, Rockford, IL, USA). For Western blot assay, 20 μg of proteins was separated by 10% SDS polyacrylamide gel electrophoresis and transferred to polyvinylidene difluoride (PVDF) membranes (GE HealthCare, Hatfield, UK), which were then treated with anti-PROX1 antibody [25], anti-phospho-MTOR (p-MTOR, ser-2448) antibody (sc-101738, Santa Cruz Biotechnology, Santa Cruz, CA, USA), anti-MTOR antibody (Ab-2448, Cusabio, Houston, TX, USA), anti-β-actin antibody (sc-47778, Santa Cruz Biotechnology, CA), appropriate secondary antibodies, and enhanced chemiluminescence detection reagent (Amersham Pharmacia Biotech, Piscataway, NJ, USA).

### 2.6. Analysis of PROX1 Ubiquitination Levels by Immunoprecipitation (IP)

Cells (1 × 10^6^) were plated in 100 mm dishes, treated with or without rapamycin (10 nM) for 24 h, and incubated with MG132 for an additional 6 h. Total proteins were extracted using NETN buffer (100 mM NaCl, 20 mM Tris-Cl [pH 8.0], 0.5 mM EDTA, 0.5% Nonidet P-40), and incubated with rabbit-IgG (1 µg; sc-2027, Santa Cruz Biotechnology) or anti-PROX1 antibody (1 µg) overnight at 4 °C, while appropriate amounts of extracted proteins were used as input controls. Agarose-conjugated protein G beads (protein G-agarose, GE-Health Care, Stockholm, Sweden) were then added and incubated for 2 h at 4 °C. Following centrifugation for 4 min at 3000 rpm, the protein G-agarose beads were washed four times with 1 mL of lysis buffer and then boiled in protein loading buffer for 5 min. Supernatants were subjected to Western blot analysis; ubiquitination was detected using an anti-ubiquitin antibody (sc-9133, Santa Cruz Biotechnology) and relative ubiquitination levels were determined using Image J.

### 2.7. Animal Studies

Animal experimental protocols were preapproved by the Institutional Animal Care and Use Committee (IACUC approval number: SCH19-0032). The seven-week-old BALB/c nude mice (male) used in this study were provided by Saeronbio (Korea). Huh7 cells were suspended in serum-free DMEM media (1 × 10^8^/mL) and inoculated subcutaneously into both flanks of each mouse (100 µL each). In the first experiment, rapamycin was injected before tumors formed (40 days after inoculation). In the second, rapamycin was injected after tumor volumes reached 100 mm^3^. Mice were treated with vehicle (5% PEG, 5% Tween-80 in PBS) or rapamycin (4 mg/kg, i.p.) daily for 14 days [25,26,27]. Tumor volumes were calculated using the following formula: V = L × W^2^/2, where V = volume (mm^3^), L = largest diameter (mm), and W = smallest diameter (mm). After CO_2_ euthanasia, tumors were extracted and fixed with 4% paraformaldehyde (PFA), following hematoxylin and eosin (H&E) staining or immunohistochemical analyses for detecting PROX1 expression.

### 2.8. Histology and Immunohistochemistry

Fixed xenograft tissues were dehydrated in an alcohol series, treated with xylene, fused with molten paraffin infusion on an automated processor, followed by paraffin-embedding, and sectioned. Immunohistochemistry was performed using a polyclonal antibody against PROX1 [25]. Sections were viewed and photographed under microscope equipped with a charge-coupled digital camera (Leica DMi8, Wetzlar, Germany). PROX1 expression was quantified using Image J, and the results are reported as mean numbers of positive pixels/tumor area.

### 2.9. Statistical Analysis

Experimental data were analyzed using GraphPad Prism 5.0 (GraphPad Software Inc., La Jolla, CA. The results are presented as the mean ± SDs of at least three independent experiments. One-way analysis of variance and Tukey’s test were used to determine the significances of differences, and statistical significance was accepted for *p*-values < 0.05 (* *p* < 0.05; ** *p* < 0.01; *** *p* < 0.001).

## 3. Results

### 3.1. The Proliferation of Huh7 or Hep3B Cells Was Inhibited by Rapamycin

**Figure 1:**
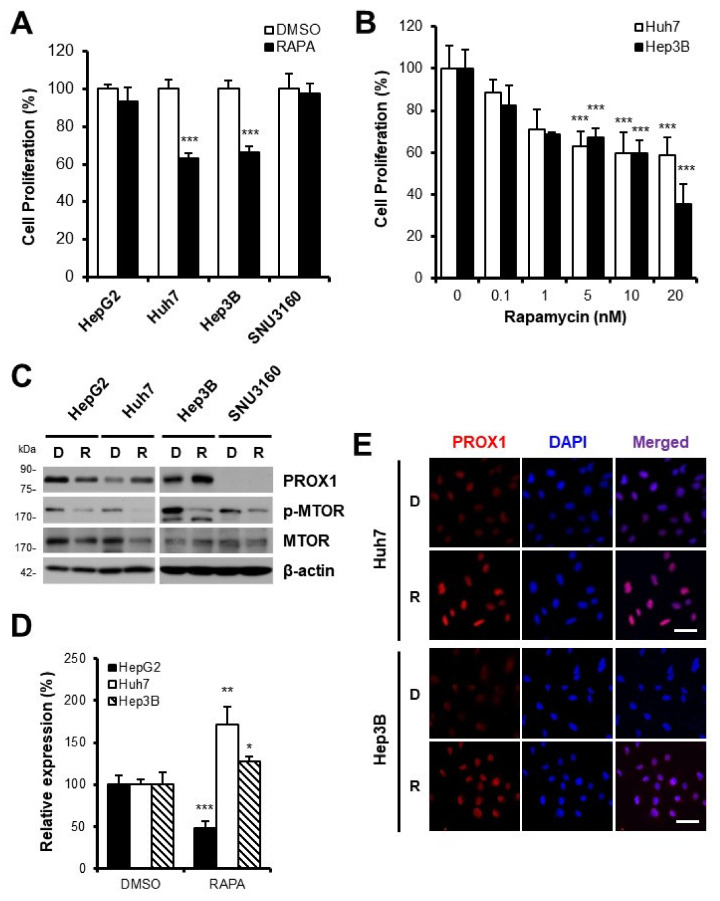
Effect of MTOR inhibition by rapamycin on HCC cell proliferation. Four different HCC cell lines (HepG2, Huh7, Hep3B and SNU3160) were cultured in conditional media (1% FBS) with or without rapamycin (RAPA). Proliferation of Huh7 or Hep3B cells was inhibited by 10 nM rapamycin for 48 h (**A**). Proliferation of Huh7 or Hep3B cells was regulated by rapamycin in a dose-dependent manner (within 0.1~20 nM) (**B**). PROX1 expression was increased by rapamycin in Huh7 or Hep3B cells. The inhibitory effect of rapamycin was confirmed by detecting the phosphorylated form of MTOR (p-MTOR). MTOR and β-actin were detected as quantitative controls (**C**). Relative expression of PROX1 were analyzed image J (**D**). PROX1 expression was also assessed by immunofluorescence microscopy (**E**). Cell nuclei were observed by DAPI staining. D: DMSO-treated cells, R: rapamycin-treated cells. Scale bars represent 100 μm. * *p* < 0.05; ** *p* < 0.01, *** *p* < 0.001.

To investigate the anti-cancer effect of rapamycin, the proliferation of HCC cell lines, Huh7, Hep3B, HepG2, or SNU3160, was measured after rapamycin treatment. Rapamycin significantly reduced the proliferation of Huh7 or Hep3B, but did not affect HepG2 or SNU3160 cells (Figure 1A), which suggests that rapamycin was not effective on all types of HCC. Rapamycin decreased the proliferation of Huh7 or Hep3B cells in a dose-dependent manner (Figure 1B). Further studies were conducted by selecting a 10 nM concentration in which the inhibitory effect on cell proliferation was confirmed in the pilot test results using various concentrations of rapamycin. 

Next, to investigate the effect of rapamycin on PROX1, all four HCC cells were treated with rapamycin, and PROX1 expression was assessed. The results from both Western blot and immunofluorescence analyses verified that rapamycin increased PROX1 expression in Huh7 or Hep3B, but not in HepG2 or SNU3160 cells, which was in line with the anti-proliferative effects of rapamycin (Figure 1C–E). On the other hand, PROX1 expression was not affected by rapamycin in HepG2 cells and not even detected in SNU3160 cells (Figure 1C,D). These results show that rapamycin increased PROX1 expression and suppressed the proliferation of Huh7 or Hep3B cells.

### 3.2. PROX1 Expression in Huh7 or Hep3B Cells Was Increased by Rapamycin

**Figure 2:**
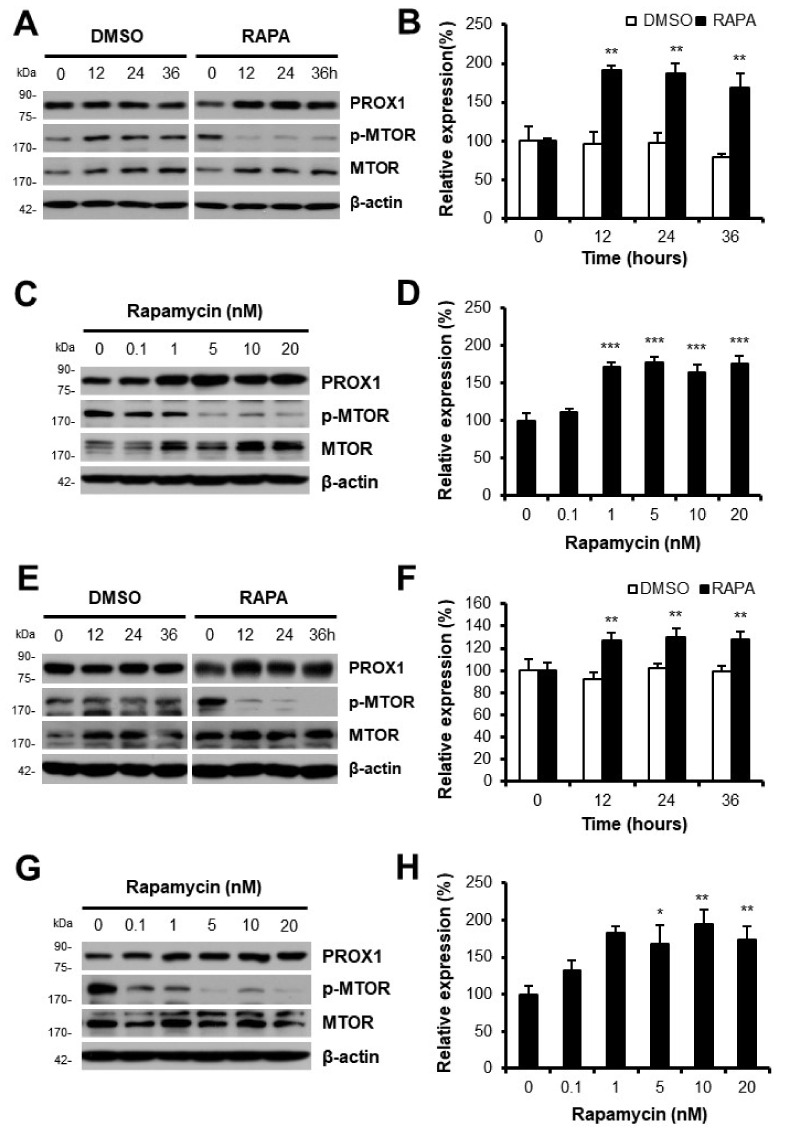
PROX1 expression was increased by rapamycin in Huh7 or Hep3B cells. PROX1 expression was elevated in Huh7 (**A**) or Hep3B cells (**E**) after 12~36 h of rapamycin treatment. The inhibitory effect of rapamycin was confirmed by detecting the phosphorylated form of MTOR (p-MTOR). MTOR and β-actin were detected as quantitative controls. The relative analysis comparing PROX1 expression with β-actin shows that PROX1 expression was significantly up-regulated by rapamycin in Huh7 (**B**) or Hep3B cells (**F**). Western blot analysis was performed after Huh7 (**C**) or Hep3B cells (**G**) were treated with rapamycin at concentrations of 0.1~20 nM. Rapamycin activity was confirmed by detecting p-MTOR. MTOR and β-actin were measured as quantitative controls. PROX1 expression in Huh7 (**D**) or Hep3B cells (**H**) was analyzed and compared with β-actin. * *p* < 0.05; ** *p* < 0.01; *** *p* < 0.001.

Next, the effect of rapamycin on PROX1 expression in Huh7 or Hep3B cells was measured. Although PROX1 expression was not affected by vehicle alone (DMSO), it was significantly enhanced from 12 h after rapamycin treatment. Phosphorylated MTOR (p-MTOR) was also detected to confirm the inhibition of MTOR by rapamycin (Huh7, Figure 2A,B; Hep3B, Figure 2E,F). PROX1 expression in cells exposed to rapamycin was increased from 1 nM of rapamycin (Huh7, Figure 2C,D; Hep3B, Figure 2G,H). These results show that PROX1 expression was increased by rapamycin in Huh7 or Hep3B cells in a time- and dose-dependent manner.

### 3.3. Increases in PROX1 Expression by Rapamycin Were Due to an Increase in Protein Half-Life

**Figure 3:**
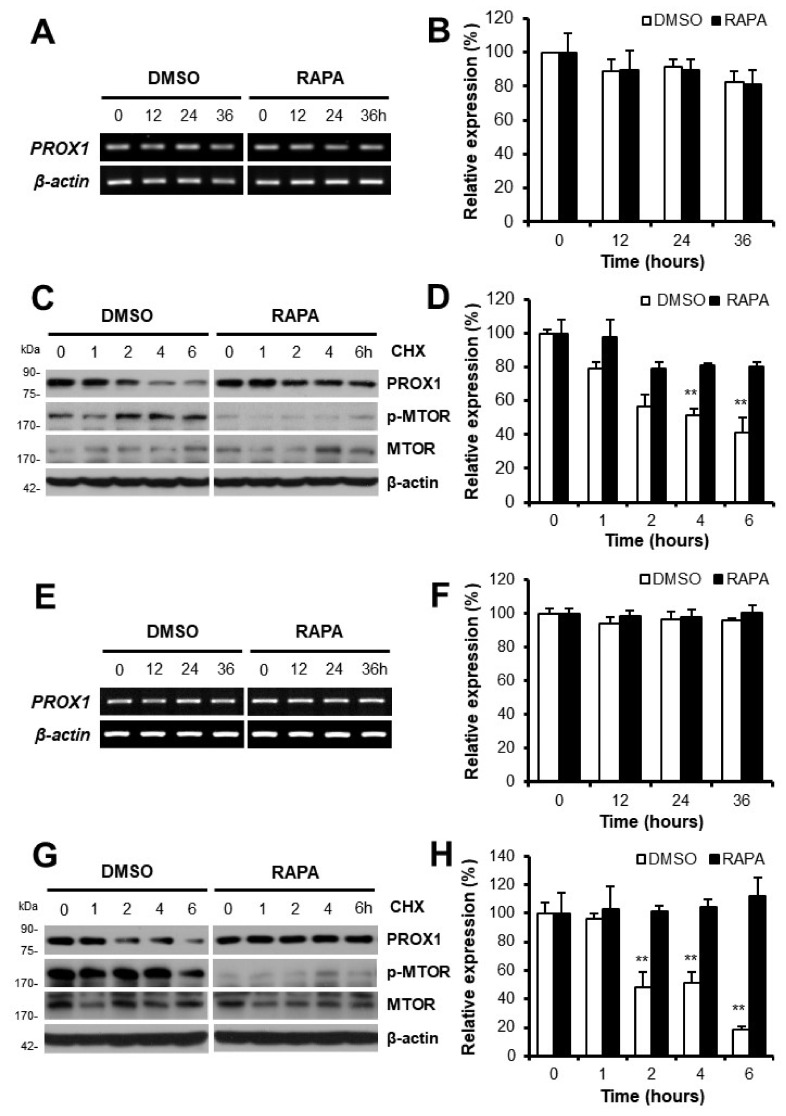
Rapamycin increased the intracellular half-life of PROX1 protein. The mRNA expression of PROX1 was analyzed by semi-quantitative RT-PCR using total RNAs prepared from Huh7 (**A**) or Hep3B cells (**E**) treated with or without rapamycin. Quantitative RT-PCR results showed that the expression of PROX1 mRNA in Huh7 (**B**) or Hep3B cells (**F**) was not affected by rapamycin. The half-life of PROX1 protein in Huh7 (**C**) or Hep3B cells (**G**) was measured by treating cells with or without rapamycin for 24 h and then exposing them to cycloheximide (50 µg/mL). Phosphorylated MTOR (p-MTOR) was detected to monitor the inhibitory effect of rapamycin. MTOR and β-actin were measured as quantitative controls. Relative analysis showed that PROX1 expression in Huh7 (**D**) or Hep3B cells (**H**) began to decrease after 2 h of cycloheximide treatment in DMSO-treated control cells, but it was maintained after rapamycin treatment for up to 6 h. ** *p* < 0.01. DMSO: DMSO-treated cells, RAPA: rapamycin-treated cells.

After verifying the upregulation of PROX1 by rapamycin in Huh7 or Hep3B cells, we explored the underlying mechanisms. Initially, we examined whether this was due to transcription or protein synthesis. We found that rapamycin did not affect the expression of PROX1 mRNA in Huh7 or Hep3B cells by semi-quantitative RT-PCR (Huh7, Figure 3A; Hep3B, Figure 3E) or quantitative RT-PCR (Huh7, Figure 3B; Hep3B, Figure 3F). We then used cycloheximide (CHX) to examine the effect of rapamycin on levels of PROX1 protein by monitoring its half-life. Interestingly, although PROX1 levels began to decrease after 2 h of CHX treatment in control cells (DMSO), levels were maintained after CHX treatment for up to 6 h in rapamycin-treated Huh7 or Hep3B cells (Figure 3C,D; Figure 3G,H). These results demonstrate that enhanced PROX1 expression in rapamycin-treated Huh7 or Hep3B cells was due to increased protein stability, not increased mRNA expression.

### 3.4. PROX1 Ubiquitination Was Decreased by Rapamycin 

**Figure 4:**
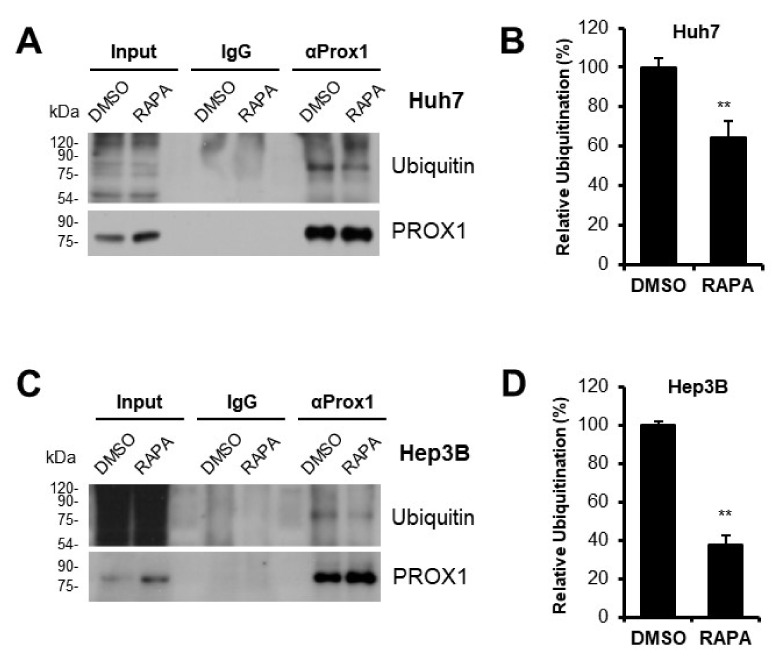
PROX1 ubiquitination was downregulated by rapamycin. Immunoprecipitation of PROX1 proteins was used for detection of ubiquitination. PROX1 ubiquitination was decreased after treatment with rapamycin for 24 h and an additional incubation with MG132 (15 µM) for 6 h in Huh7 (**A**) or Hep3B (**C**) cells. Relative analysis showed that PROX1 ubiquitination was suppressed by rapamycin (**B**,**D**). ** *p* < 0.01. DMSO: DMSO-treated cells, RAPA: rapamycin-treated cells.

To understand why PROX1 protein levels were increased by rapamycin, we investigated the changes in its ubiquitination. Immunoprecipitation with anti-PROX1 antibody showed the ubiquitination of PROX1 was downregulated by rapamycin in Huh7 (Figure 4A) and Hep3B cells (Figure 4C). As compared with untreated cells, PROX1 ubiquitination was decreased by ~40% in Huh7 cells (Figure 4B) and by over 60% in Hep3B cells by rapamycin (Figure 4D). These data show that the higher levels of PROX1 in rapamycin-treated Huh7 or Hep3B cells were due to the suppression of PROX1 ubiquitination.

### 3.5. PROX1 Played a Key Role in the Anti-Proliferative Effect of Rapamycin on Huh7 or Hep3B Cells

**Figure 5:**
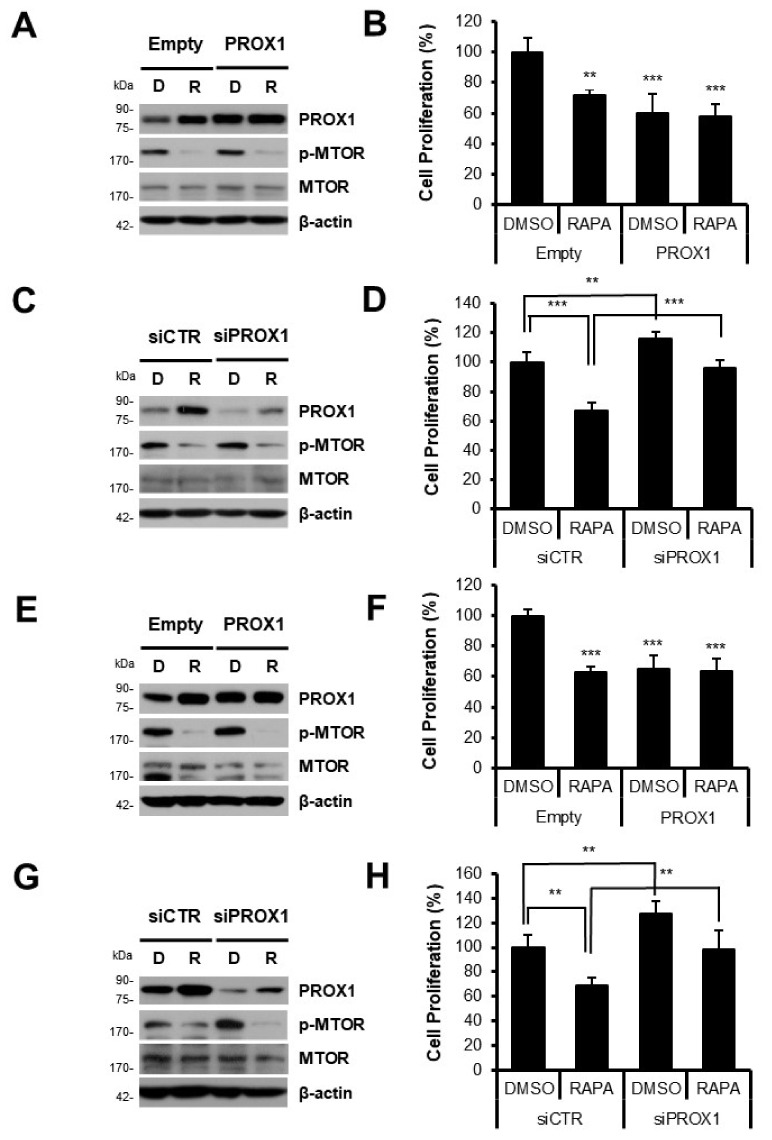
PROX1 played a key role in the anti-proliferative effect of rapamycin. Huh7 (**A**) or Hep3B cells (**E**) were transfected with a plasmid expressing human PROX1 or an empty plasmid, and 24 h later were treated with or without rapamycin for an additional 24 h. Western blot analysis showed that PROX1 expression was increased by the over-expression system and by rapamycin, and markedly by rapamycin treatment plus PROX1 over-expression. D: cells treated with DMSO, R: cells treated with rapamycin. The effect of PROX1 on the proliferation of Huh7 (**B**) or Hep3B cells (**F**) was also investigated using the over-expression system. After 24 h of transfection, cells were treated with or without rapamycin for an additional 48 h. Cell proliferation was inhibited by rapamycin and by PROX1 over-expression. Huh7 (**C**) or Hep3B cells (**G**) were transfected with siRNA targeting PROX1 mRNA (siPROX1) or control siRNA (siCTR). After 24 h, cells were treated with or without rapamycin for an additional 24 h. Western blot analysis clearly showed the knock-down effect of siPROX1. The inhibitory effect of rapamycin was confirmed by determining phosphorylated MTOR (p-MTOR). MTOR and β-actin were detected as quantitative controls. To investigate the effect of siPROX1 on cell proliferation, Huh7 (**D**) or Hep3B cells (**H**) were transfected with siPROX1 and treated with or without rapamycin for an additional 48 h. siPROX1treatment was found to increase the proliferation of Huh7 or Hep3B cells and to decrease the anti-proliferative effect of rapamycin. **, *p* < 0.01; ***, *p* < 0.001. DMSO: DMSO-treated cells, RAPA: rapamycin-treated cells.

To confirm the role of PROX1 in HCC proliferation, we used a PROX1 over-expression system to mimic the effect of rapamycin. Western blot analysis showed that PROX1 expression was substantially increased in both rapamycin-treated and PROX1 over-expressed cells, which confirmed the effectiveness of the over-expression system (Huh7, Figure 5A; Hep3B, Figure 5E). Notably, the proliferation of Huh7 or Hep3B cells was significantly reduced by PROX1 over-expression and by rapamycin treatment (Huh7, Figure 5B; Hep3B, Figure 5F). To investigate further the functional roles of PROX1 in HCC proliferation, we used siRNA targeting PROX1 mRNA (siPROX1). Western blot analysis confirmed that siPROX1 effectively inhibited PROX1 expression (Figure 5C,G). Next, we tested whether siPROX1 could inhibit the proliferation of cells in the presence or absence of rapamycin and found that the anti-proliferative effect of rapamycin was significantly suppressed by siPROX1 (Huh7, Figure 5D; Hep3B, Figure 5H). Collectively, these results show that PROX1 played a critical role in the anti-proliferative effect of rapamycin on Huh7 or Hep3B cells.

### 3.6. Xenograft Tumor Growth Was Suppressed and PROX1 Expression Was Increased by Rapamycin In Vivo

**Figure 6:**
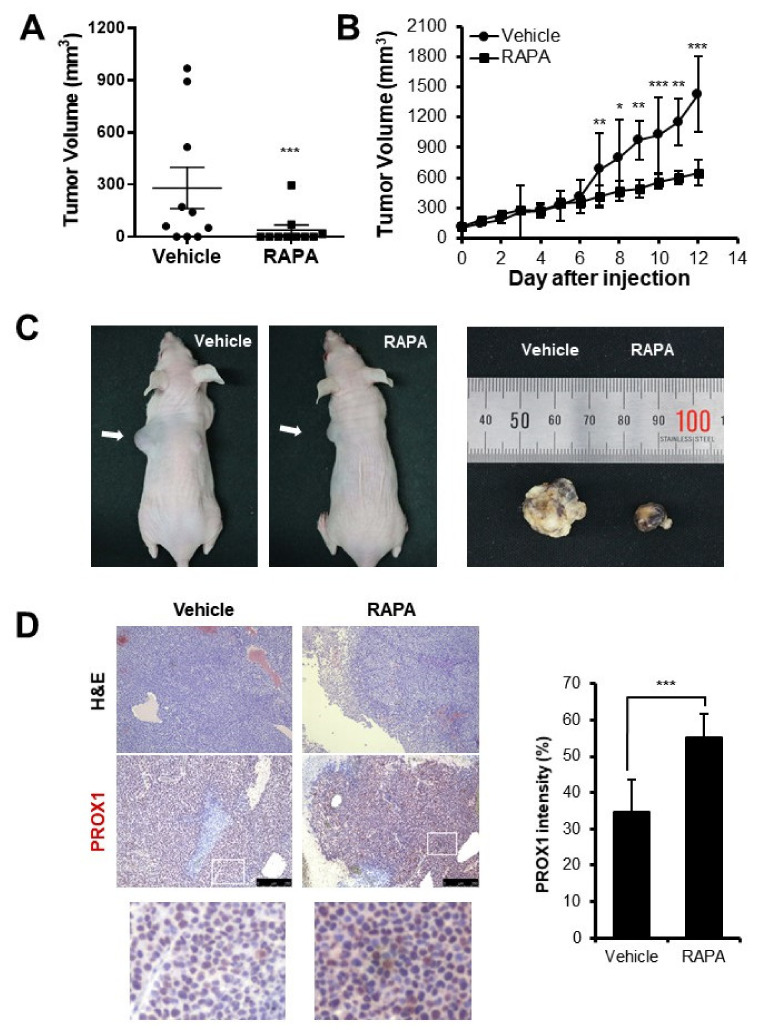
The effect of rapamycin on Huh7 xenograft growth and intratumoral PROX1 levels. Huh7 cells (1 × 10^7^) were inoculated into the flanks of seven-week-old BALB/c nude mice. Tumor growth was effectively suppressed by rapamycin injected daily (at 4 mg/kg, n = 10, respectively) for 14 days before xenograft tumor formation (**A**). In the second experiment, rapamycin was injected for 14 days, after the tumors reached 100 mm^3^ (n = 5, respectively). Rapamycin suppressed tumor growth (**B**,**C**) and increased PROX1 levels (**D**). * *p* < 0.05; ** *p* < 0.01; *** *p* < 0.001.

To evaluate the effect of PROX1 expression in human HCC in vivo, Huh7 cells were inoculated into the flanks of nude mice for xenograft study. In the first experiment, rapamycin was administered daily for 14 days from the 40th day when the tumor began to grow. It was found that Huh7 xenograft did not develop (n = 10, Figure 6A). In the second experiment, we injected rapamycin or vehicle daily for 14 days after the tumor volumes reached 100 mm^3^ and found that tumor volumes were significantly smaller in the rapamycin-treated mice than in vehicle controls (average 647 mm^3^ vs. 1425 mm^3^, n = 5, respectively, Figure 6B,C). Subsequent immunohistochemical analyses of tumors showed that rapamycin increased PROX1 expression (Figure 6D). These in vivo results show that rapamycin suppressed Huh7 xenograft growth and increased intratumoral PROX1 expression.

## 4. Discussion

HCC is characterized by several genetic changes that affect multiple signal pathways and result in the uncontrolled proliferation of hepatocytes [28]. Rapamycin inhibits MTOR and has been widely used to prevent the rejection of transplanted organs [16]. In addition to being used as an immunosuppressant, the clinical applications of rapamycin have been extended to cancer treatment. Recently, MTOR-specific inhibitors were examined as potential anti-cancer agents and showed anti-cancer activity in several patients with advanced HCC [29]. However, a clinical trial (EVOLVE-1) of the MTOR inhibitor, everolimus, showed that it did not improve overall survival in advanced HCC patients unresponsive or intolerant to sorafenib [30]. Therefore, a suitable chemotherapy agent that specifically targets a molecule required by HCC is urgently required to enable the development of personalized targeted therapy.

Here, we investigated the anti-proliferative effect of rapamycin, an MTOR inhibitor, on four different HCC cell lines, Huh7, Hep3B, HepG2, and SNU3160. SNU3160 cells are WHO grade G2 (moderately differentiated), HBV negative, and PROX1-negative. Interestingly, the anti-proliferative effects of rapamycin were only apparent in Huh7 or Hep3B (Figure 1A), which was probably owing to their heterogeneous origins. Indeed, it is well known that HCC has a heterogeneous etiology, and thus only some HCC patients benefit from sorafenib treatment. The anti-proliferative effect of MTOR inhibition on Huh7 or Hep3B cells was confirmed with temsirolimus, another MTOR inhibitor (data not shown).

During our investigation to determine whether underlying molecular mechanisms might explain these results, it was observed that the anti-proliferative effect appeared only in cell lines where PROX1 protein increased after rapamycin treatment Although rapamycin treatment significantly and time- and dose-dependently increased PROX1 expression in Huh7 or Hep3B cells (Figure 2), interestingly, rapamycin suppressed PROX1 expression in HepG2 cells (Figure 1C,D). We previously reported the inhibitory effect of rapamycin on PROX1 expression, and resultant triglyceride accumulation in hepatocytes [25]. Notably, PROX1 protein was not detected in SNU3160 cells (Figure 1C). In addition, cell proliferation was not affected by high concentrations of rapamycin, but was inhibited by PROX1 over-expression in these two cell lines (data not shown).

As there are few reports showing the relationship between the MTOR signal and PROX1, further investigation is needed to identify the mechanisms by which the MTOR signal affects PROX1 expression and to determine the causes of different effects of rapamycin on PROX1 expression among HCC cell lines. To understand the upregulation of PROX1 by MTOR inhibition, changes in its mRNA expression were first investigated. Although the mRNA expression of PROX1 was not affected, the half-life of PROX1 protein was enhanced by rapamycin in Huh7 or Hep3B cells (Figure 3), which was achieved by reduced PROX1 ubiquitination (Figure 4). The upregulation of PROX1 protein might be related to the anti-proliferative effect of rapamycin in Huh7 or Hep3B cells. Because there are few studies reporting the process of PROX1 ubiquitination, further studies are required to identify and investigate factors related to PROX1PROX1 ubiquitination, and further studies are required to identify and investigate factors related to PROX1 ubiquitination, such as E3 ligase.

To verify the participation of PROX1 in the anti-proliferative effect of rapamycin, PROX1 over-expression was used to mimic the effect of rapamycin in Huh7 or Hep3B cells. Cell proliferation was downregulated by PROX1 over-expression in both cell lines in a manner similar to that of rapamycin (Huh7, Figure 5B; Hep3B, Figure 5F). Interestingly, the loss-of-function experiment using PROX1 siRNA showed that the anti-proliferative effect of rapamycin was abrogated by PROX1 knock-down (Figure 5D,H). PROX1 protein levels were not affected by rapamycin in Huh7 and Hep3B cells transfected with the PROX1-expressing vector, which means that the effect of MTOR inhibition on PROX1 expression was diminished by the overexpression system.

To check the anti-cancer effect of PROX1 and MTOR inhibition in vivo, Huh7 cells were inoculated into the flanks of nude mice. In the first experiment, rapamycin was injected into mice before xenografts formed, and as expected, tumor development was suppressed well by rapamycin (Figure 6A). In the second experiment, rapamycin was injected when the tumors volume exceeded 100 mm^3^. Again, tumor growth was suppressed by rapamycin (Figure 6B,C), and immunohistochemical analyses showed intratumoral PROX1 levels were increased by rapamycin (Figure 6D). These results indicate that increased PROX1 expression was related to the anti-proliferative effect of rapamycin on HCC.

In conclusion, we found that rapamycin, an MTOR inhibitor, reduced the proliferation of Huh7 or Hep3B cells by increasing the half-life of PROX1 protein. Functional studies using an over-expression, or a knock-down, system showed that PROX1 critically mediates the anti-proliferative effect of rapamycin on Huh7 or Hep3B cells. Furthermore, our xenograft study revealed that the growth of Huh7 xenografts was suppressed and that PROX1 expression was increased by rapamycin. Together, these results suggest that PROX1 plays a critical role in the anti-proliferative effect of rapamycin on HCC and that the upregulation of PROX1 by an MTOR inhibitor might be a useful molecular marker for predicting the efficacy of its anti-cancer effect on HCC cells.

## Data Availability

Not applicable.
